# Internal oligo(dT) priming introduces systematic bias in bulk and single-cell RNA sequencing count data

**DOI:** 10.1093/nargab/lqac035

**Published:** 2022-05-25

**Authors:** Marek Svoboda, H Robert Frost, Giovanni Bosco

**Affiliations:** Quantitative Biomedical Sciences Program, Geisel School of Medicine, Dartmouth College, Hanover, NH 03755, USA; Department of Biomedical Data Science, Geisel School of Medicine, Dartmouth College, Hanover, NH 03755, USA; Molecular and Systems Biology Program, Geisel School of Medicine, Dartmouth College, Hanover, NH 03755, USA

## Abstract

Significant advances in RNA sequencing have been recently made possible by using oligo(dT) primers for simultaneous mRNA enrichment and reverse transcription priming. The associated increase in efficiency has enabled more economical bulk RNA sequencing methods and the advent of high-throughput single-cell RNA sequencing, already one of the most widely adopted methods in transcriptomics. However, the effects of off-target oligo(dT) priming on gene expression quantification have not been appreciated. In the present study, we describe the extent, the possible causes, and the consequences of internal oligo(dT) priming across multiple public datasets obtained from various bulk and single-cell RNA sequencing platforms. To explore and address this issue, we developed a computational algorithm for RNA counting methods, which identifies the sequencing read alignments that likely resulted from internal oligo(dT) priming and removes them from the data. Directly comparing filtered datasets to those obtained by an alternative method reveals significant improvements in gene expression measurement. Finally, we infer a list of human genes whose expression quantification is most likely to be affected by internal oligo(dT) priming and predict that when measured using these methods, the expression of most genes may be inflated by at least 10% whereby some genes are affected more than others.

## Graphical Abstract

**Graphical Abstract:**
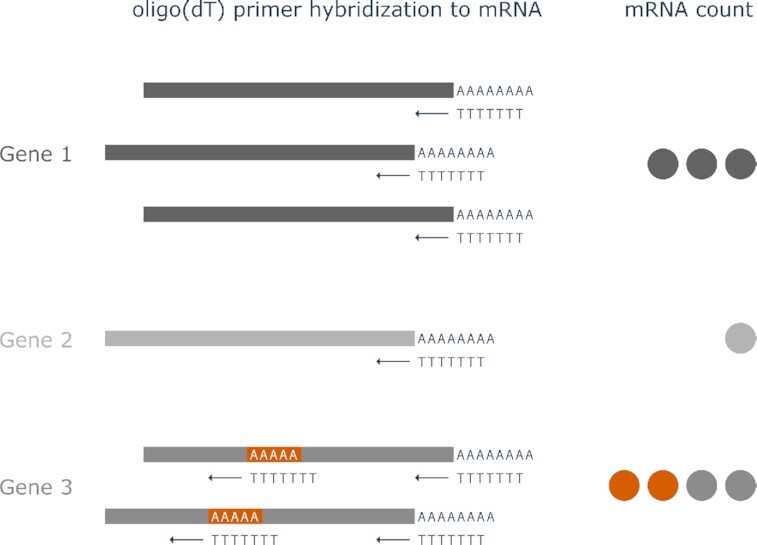
In RNA sequencing methods that use oligo(dT) primers, hybridization onto genome-encoded adenine single nucleotide repeats, in addition to poly(A) tails, leads to overinflated mRNA counts for genes containing these repeats.

## INTRODUCTION

Next-generation sequencing (NGS) of RNA is one of the most commonly used methods to measure gene expression in tissues as well as single cells, yielding readily quantifiable information about the relative levels of protein production as a proxy to cellular activity.

Oligo(dT) probes have been used in RNA sequencing to simultaneously enrich for poly(A) tail-containing mRNAs and prime the reverse transcription to cDNA. This highly efficient approach is especially useful for gene expression quantification using very low abundance input RNA - for example, from individual cells (1). For this reason, oligo (dT) priming is employed by virtually all existing high-throughput single-cell RNA sequencing (scRNA-seq) methods (e.g. Drop-seq, inDrop, 10X, Seq-well, sci-RNA-seq) (2–6), some of the low-throughput ones (e.g. Smart-seq, CEL-seq) (7,8), and even some cost-effective bulk RNA sequencing methods (e.g. QuantSeq, TagSeq, 3′Pool-seq) (9–11).

Most of these methods aim to quantify gene expression by counting the number of mRNA molecules that originate from their respective genes using the underlying assumption that oligo(dT) priming only takes place at the 3′-poly(A) tails. The number of priming events is therefore assumed to correspond to the number of original molecules of mRNA before any subsequent PCR amplification takes place. However, this assumption is only correct for transcripts that do not contain additional, internal oligo(dT) priming sites.

**Figure 1.**
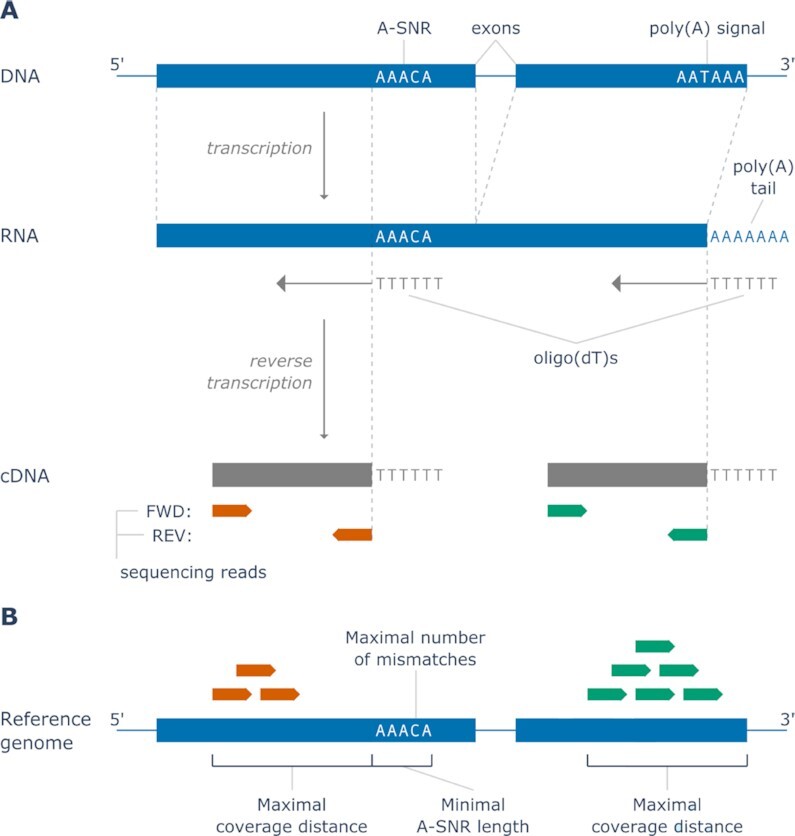
Oligo(dT) priming-based RNA sequencing and filtering parameters. (**A**) A simplified diagram describing the overall steps in transcription from DNA to mRNA and the subsequent process of oligo(dT) priming, reverse transcription to cDNA, and sequencing. Internal oligo(dT) priming takes place at genome-encoded A-SNRs, while terminal oligo(dT) priming takes place at post-transcriptionally added poly(A) tails. Depending on the method used, the sequencing takes place either from the 5′ (FWD methods used for mRNA quantification) or the 3′ (REV methods used for polyadenylation site detection) end of the cDNA fragment, resulting in reads in either sense or antisense direction, respectively. For that reason, each sequencing read in the REV methods is directly adjacent to the oligo(dT) priming event that gave rise to its cDNA, while in the FWD methods, the priming event and the associated sequencing read are separated by a gap of variable length given by the length of the cDNA fragment sequenced. (**B**) An illustration of FWD internal and terminal sequencing reads aligned to their respective reference gene with the parameters used in the filtering algorithm visualized. All sequencing reads aligned within the *maximal coverage distance* upstream of all A-SNRs of *minimal A-SNR length* with up to the *maximal number of mismatches* (i.e. internally primed reads, in orange) are removed from the dataset, while any sequencing reads aligned within the same *maximal coverage distance* upstream of the 3′ ends of any transcripts (i.e. terminally primed reads, in green) are always kept in the dataset. Note that the filtering algorithm was designed specifically for FWD sequencing methods, in which the variable gap between the oligo(dT) priming events and the associated sequencing read alignments in the reference genome is accounted for by the *maximal coverage distance* filtering parameter.

While the 3′-terminal poly(A) tails of mRNA are added during post-transcriptional processing of nascent mRNAs, some of the RNA molecules also contain genome-encoded poly(A) sequences (adenine single-nucleotide repeats or A-SNRs). These internal priming sites can lead to ‘off-target’ oligo(dT) hybridization, as reported previously in studies on gene identification, alternative polyadenylation, and scRNA-seq analysis (Figure 1A).

Early gene identification efforts that relied on 3′-terminal oligo(dT) priming of entire transcripts often resulted in truncated cDNAs, whereby internally hybridized oligo(dT)s initiated cDNA synthesis and simultaneously prevented extension of reverse transcription primed at a downstream poly(A) tail (12). Through this process, internal priming led to the misidentification of the extent of transcripts. Additionally, it has been suggested that internal oligo(dT) priming (i.e. onto genome-encoded A-SNRs) may even take place at a higher frequency than terminal priming (i.e. onto poly(A) tails), in a ratio of up to 3:1. This seems to be due to the fact that the relatively higher stability of oligo(dT) hybridization to the long poly(A) tails may paradoxically often lead to their hybridization with 3′ overhangs, preventing cDNA extension. The recommended solution is the use of anchored oligo(dT) primers (i.e. oligo(dT)s with 1–2 nucleotides other than T at their 3′ end), which relatively limits but does not completely prevent internal oligo(dT) priming, pointing to the need to remove these alignments from the resulting data computationally.

Similarly, in studies that focus on alternative polyadenylation, internal oligo(dT) priming may lead to the identification of false polyadenylation sites (13,14). In this context, various rules and methods have been previously used in data analysis to identify and filter out sequencing read alignments that might have resulted from internal oligo(dT) priming. Examples include identification of the following genomic features directly adjacent to the 3′ end of the alignments, suggestive that the sequencing reads originated from internal oligo(dT) priming: six continuous adenines (As) or more than seven As in a 10 nucleotide (nt) window (13), AG-runs of six or more nts or eight or more As or Gs in a 10 nt window (15), eight or more As or high A/T content (27 out of 30 bases) (16), and 12 or more adenines present in an 18 nt window (17). However, because these polyadenylation site studies use 3′ reverse sequencing reads directly adjacent to their associated priming events (‘REV,’ Figure 1A), these filtering methods are not suitable for use in RNA sequencing methods that use 5′ forward reads (i.e. the methods most frequently used for gene expression quantification), which are always separated by some genomic distance from their respective priming events (‘FWD,’ Figure 1A).

In the context of scRNA-seq, internal oligo(dT) priming has only been previously discussed for the purposes of analysis termed ‘RNA velocity’ (18). Because A-SNRs are commonly found in introns, oligo(dT) hybridization to these sequences in unspliced pre-mRNA may lead to the generation of intronic sequencing reads. Therefore, in RNA velocity, detection of intronic alignments in scRNA-seq data is used to derive changes in gene expression over time. However, the contribution of internal priming to exonic alignments, which are the ones most often used for gene expression quantification, has not been previously characterized in scRNA-seq data. Additionally, a bias in sequencing data associated with oligo(dT)s hybridizing to genomic poly(A) sequences has been previously reported in the SMART library preparation method (19), which is used in scRNA-seq to yield complete coverage across transcripts, but the impact on the measured gene expression remains unclear.

To our knowledge, internal oligo(dT) priming to A-SNRs has not been thoroughly characterized nor previously addressed in the context of the FWD sequencing methods, which are used for gene expression quantification and therefore constitute the most common use of oligo(dT) priming in RNA sequencing, including virtually all scRNA-seq methods. The fact that oligo(dT) primers may spuriously hybridize to one or more internal, genome encoded A-SNRs in addition to the targeted poly(A) tails of mRNA molecules from a given gene could lead to a higher probability or even multiplicity of detection of mRNA molecules that contain these A-SNRs. We, therefore, reasoned that internal oligo(dT) priming may lead to relative overinflation of measured mRNA molecule abundance in genes that contain genome-encoded poly(A) sequences (A-SNRs), introducing bias in the gene expression data.

We analyzed publicly available datasets obtained with various bulk and single-cell RNA sequencing methods that use oligo(dT) priming and found that all of them exhibited signs of internal priming, suggesting that this is a widely prevalent phenomenon. We subsequently developed the first algorithm to filter out the sequencing read alignments that likely resulted from internal priming in oligo(dT)-based FWD sequencing RNA count datasets. We show that removing these internally primed alignments as an additional data processing step improves the accuracy of gene expression count data. Based on these findings, we ranked human genes by their likelihood to be subject to internal oligo(dT) priming when expressed, suggesting that expression of more than half of all genes may be inflated by at least 10% but that this phenomenon does not affect all genes equally.

## MATERIALS AND METHODS

### Datasets used

All datasets used originated from previous publications by authors not associated with this study and are publicly available. For the *ERCC spike-in standards analysis*, the QuantSeq 3′ mRNA-Seq REV dataset by Wu *et al.* (20) was obtained from Gene Expression Omnibus (GEO) Accession GSE137612. Specifically, the sample ‘siGFP_noPAP_in_batch5’ (FASTQ file from Sequence Read Archive (SRA) SRR10134316) was selected due to being a control sample with the highest sequencing depth. This dataset was also used to calculate the *aggregate exonic RNA sequencing coverage* upstream of transcript 3′ ends and A-SNRs, along with the QuantSeq dataset by Ma *et al.* (21) and PBMC1 10X (v2) A dataset by Ding *et al.* (22), as described below. *Parameter optimization through iterative filtering* was carried out on the following datasets: QuantSeq 3′ mRNA-Seq FWD dateset by Ma *et al.* (21) was obtained from GEO Accession GSE116949, using the ‘Lexogen control diet 1’ sample (SRA FASTQ file SRR7510922) along with the associated random oligo priming-based ‘KAPA control diet 1’ sample (SRA FASTQ file SRR7510916). The 10X, CEL-seq2, Drop-Seq, inDrop, and Seq-well single cell (Human PBMC1 and PBMC2) and single nuclei (Mouse Cortex1) datasets by Ding *et al.* (22) were obtained along with the respective random oligo priming-based bulk datasets from GEO Accession GSE132044.

### ERCC spike-in standards analysis

Using the FASTQ files by Wu *et al.* (20), a BAM file was generated as described in the original publication except for removal of alignments mapped to genomic A-rich positions. STAR v2.7.3a was used for genome indexing and read alignment (23).

**Figure 2.**
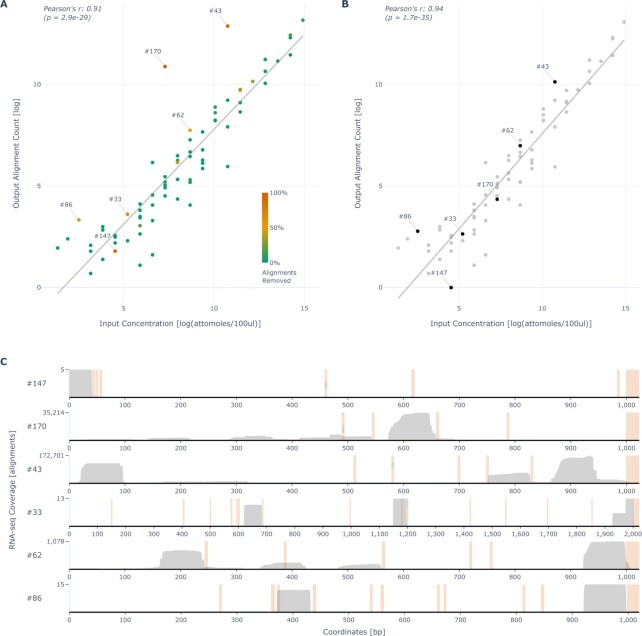
Removal of non-terminal ERCC alignments from bulk oligo(dT) priming-based data. (**A**) Correlation between the log of input ERCC concentrations and log of the respective alignment counts detected using oligo(dT)-primed bulk RNA sequencing; data by Wu *et al.* (20). ERCCs not detected are not shown. The color scale indicates the proportions of non-terminal reads (>75 bp upstream of the poly(A) tail) aligned to each respective ERCC reference. Numbers of the six ERCCs with the highest proportions of non-terminal coverage are annotated. (**B**) Correlation plot similar to that in (A) after the removal of non-terminal alignments, with the same ERCCs annotated. (**C**) The RNA sequencing coverage tracks of the six ERCCs annotated in (A) and (B). Per-basepair sequencing coverage is shown in gray with the maximum coverage indicated on the y-axis. In orange, SNRs of five or more consecutive As (including the poly(A) tails, which are synthesized as a part of the ERCCs) in each ERCC reference are highlighted (*P*-values were calculated using a two-tailed *t*-test).

Subsequently, all alignments mapped to the ERCC reference sequences were counted with a custom python script using package pysam v0.16.0.1, while alignments that mapped further than 75 bp upstream of the ERCC poly(A) tails were counted as non-terminal (Figure 2). The distance of 75 bp was selected as a conservative cutoff to signify a distinct priming event, considering that Wu *et al.* clustered any alignments within 25 bp for the purposes of their 3′ end analysis and the sequencing length used to generate this data was 75 bp. ERCCs with no alignments detected (before removal of non-terminal alignments) were removed from the data. To plot and calculate the correlations of input concentrations vs. output alignment counts, the input concentrations (originally in attomoles/ul) were multiplied by 100 to obtain attomoles/100 ul. Subsequently, for both the input concentrations and the output alignment counts, the natural log-transformation was applied after adding a pseudocount of 1. Aggregate coverage per bp along the ERCC reference sequences was also obtained using pysam.

### Genome scanning for A-SNRs

**Figure 3.**
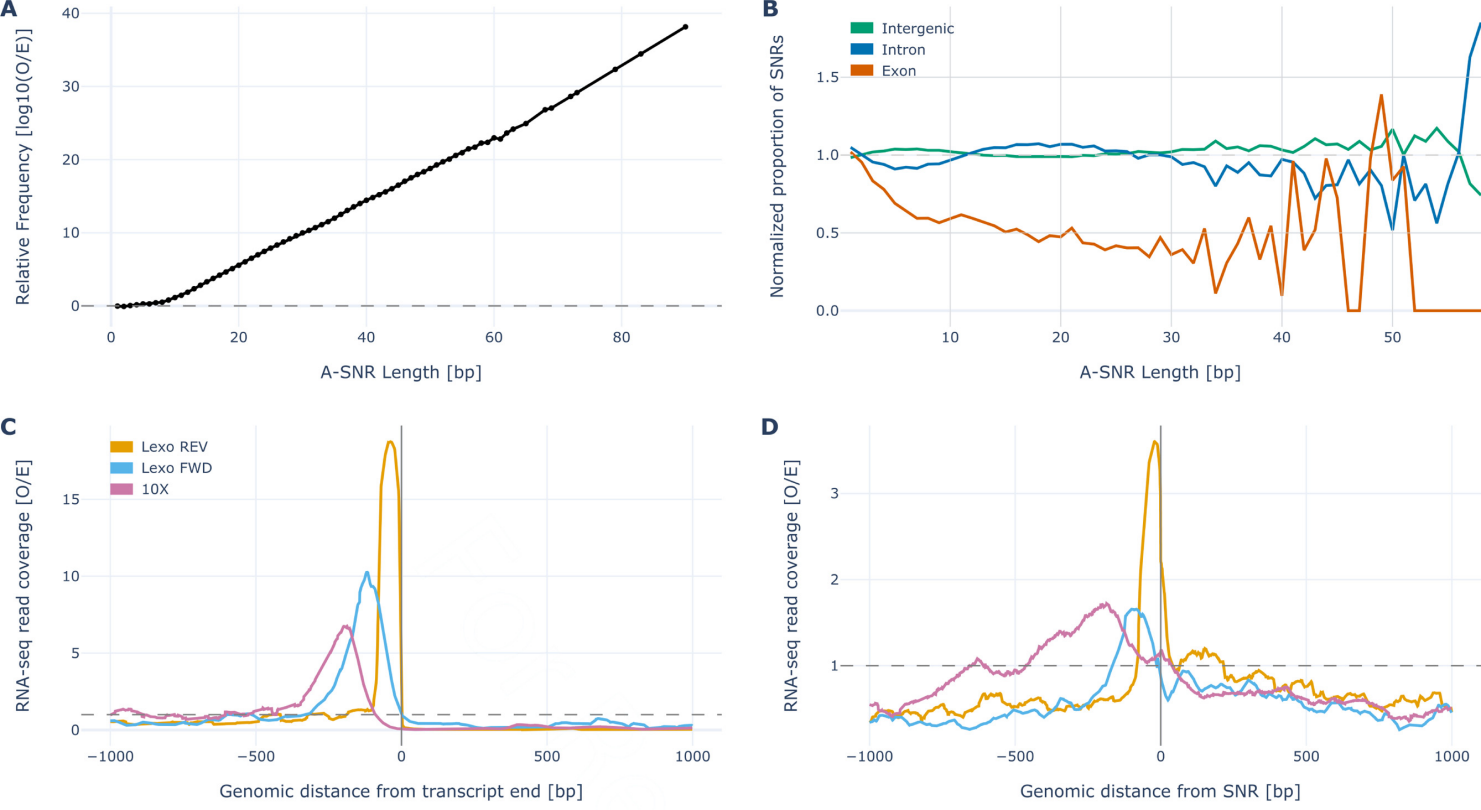
Genome encoded A-SNRs and the associated sequencing coverage. (**A**) Log_10_ of relative (observed/expected) frequency of A-SNRs by length across the entire human genome reference. In the gray dashed line, the expected frequency represents the probability that A-SNRs of a given length be found by pure chance. (**B**) The relative enrichment of A-SNRs of each length by their genomic regions, normalized by the respective proportions of the human genome thus annotated. In the gray dashed line, the expected normalized proportion of A-SNRs for each region represents a random allocation of A-SNRs across the genome. SNR lengths represented by fewer than 10 SNRs (i.e. longer than 58 bp) were truncated. (**C**) Normalized aggregate stranded (i.e. on the sense strand for both FWD methods and antisense strand for the REV method) RNA sequencing coverage in the vicinity of all non-overlapping transcript 3′ ends, which are aligned at ‘0 bp’ in the sense orientation. (**D**) Normalized aggregate stranded RNA sequencing coverage in the vicinity of all exonic A-SNRs of five nucleotides or longer, whose starts (5′ ends) are aligned at ‘0 bp’ in the sense orientation. In (C) and (D), sequencing coverage from datasets by Wu *et al.* (‘Lexo REV’) (20), Ma *et al.* (‘Lexo FWD’) (21) and Ding *et al.* (‘10X’) (22) is depicted in the vicinity of 31 031, 45 205 and 31 031 transcript 3′ ends (C) and 235 318, 203 253, and 235 068 A-SNRs of five nucleotides or longer (D), respectively. The gray dashed line represents the expected coverage, i.e. aggregate exonic sequencing coverage in the dataset divided by the total length of all non-overlapping exons in the respective genome.

We used the GRCh38 v3.0.0 Cell Ranger reference (human, Figure 3A, B), mm10 v3.0.0 Cell Ranger reference (mouse, Supplementary Figure S1A), Dmel_Release_6 FlyBase reference (fruit fly, Supplementary Figure S1B) (24), and Os-Nipponbare-Reference-IRGSP-1.0 reference (rice, Supplementary Figure S1C) (25) for genome scanning and assignment of genomic annotations. The reference genomes were scanned for A-SNRs on (as well as T-SNRs, i.e. A-SNRs on the opposite strand) with a custom python script using Biopython v1.74 Bio.SeqIO package (26) and relative (observed/expected) A-SNR frequency was calculated as described by Murray (27). Genomic annotations were extracted using python package gffutils v0.10.1. The proportions of SNRs at each length assigned to their respective genomic annotations were normalized by the total proportion of the genome with this annotation, as follows: genomic regions annotated as ‘exon’ in at least one transcript were considered exonic, the remaining non-exonic genomic regions covered by at least one transcript (annotated as ‘transcript’ in most references, or as ‘mRNA’ in the fruit fly reference) were considered intronic, and the remaining genomic regions were considered intergenic.

### Aggregate exonic RNA sequencing coverage

Each of the datasets was processed as described by the authors in the respective original publications; datasets by Ding *et al.* (22) were always processed using their scumi pipeline to ensure a fair comparison between different library preparation methods. The respective reference annotations were scanned for 3′ ends of non-overlapping transcripts, the reference sequences were scanned for A-SNRs, and the dataset BAM files were used to calculate the surrounding RNA sequencing coverage (Figure 3C, D and Supplementary Figure S2). Per-base sequencing coverage was subsequently normalized by the expected (mean) per-base coverage across all exons (i.e. total exonic coverage across the entire genome divided by the number of non-overlapping bps annotated as exons) and divided by the number of potential priming events (transcript 3′ ends or A-SNRs).

### Python algorithm to filter out internal alignments

We designed a custom python algorithm that uses Biopython’s Bio.SeqIO, gffutils and pysam packages to scan a given reference genome for A-SNRs of given minimal length (the *minimal A-SNR length* parameter) with a given maximal number of mismatches (i.e. any non-A bases; the *maximal number of mismatches* parameter), to remove all non-terminal alignments in a given dataset (binary alignment map (BAM) file) that are found up to a given coverage distance upstream of each A-SNR identified, but not within the same distance of a 3′ end of any annotated transcript (the *maximal coverage distance* parameter, Figure 1B).

Specifically, the algorithm does so by carrying out the following sequence of steps:

- Scan the reference genome annotation (a GTF/GFF file) for all transcripts and save those that have any exonic coverage associated with them in the sequencing dataset (a BAM file).
- Protect the 3′-terminal segments of all covered transcripts up to exonic (i.e. not counting introns) length equal to the *maximal coverage distance*. Any sequencing reads aligned within these protected segments will be kept in the dataset.
- Scan the reference genome sequence (a FASTA file) to identify all A-SNRs whose length is equal to or greater than the *minimal A-SNR length*, with the *maximal number of mismatches* allowed. No mismatches are allowed in the first or last bp of the A-SNR.
- Identify internal priming segments up to the *maximal coverage distance* upstream of these A-SNRs and overlapping non-terminal exonic portions of all covered transcripts.
- Remove all alignments in the dataset (a BAM file) that overlap with these internal priming segments.

It is also possible to run this algorithm with the option to include the intronic annotations and coverage (as was done in the case of single nuclei RNA sequencing data filtering). When *minimal A-SNR length* is set to 0 (or ‘None’), this algorithm removes all non-terminal alignments—i.e. all reads that are not aligned within the segments protected in step 2, regardless of their association with A-SNRs.

### Parameter optimization through iterative filtering

Each of the oligo(dT) priming-based datasets studied was processed as described in the respective original publication except for additionally filtering thus generated BAM files, as described below, before further processing the data.

Using the algorithm described in the previous section and varying the three input parameters, we repeatedly filtered each of the oligo(dT) priming-based datasets studied to maximize the correlation with the random oligo priming-based gold standard dataset obtained from the same biological sample (the correlations were calculated as described in the respective original publications). Due to the limitations of the computational resources available, instead of testing all the possible input parameter combinations, we carried out the filtering iteratively, using the set of parameters in the vicinity of those that previously yielded the highest correlation, assuming that the underlying function was generally concave with one global maximum and no local maxima.

We selected a conservative set of starting filtering parameters based on our observations (Figure 3C, D and Supplementary Figure S2), such that the optimal values were expected to be found within the ranges tested, as follows:

*Maximal coverage distance*:   100, 300, 500, 700, 900

*Minimal A-SNR length*:     0, 4, 8, 12, 16

The *minimal A-SNR length* of ‘0’ indicates that all non-terminal alignments were removed, regardless of their association with any A-SNRs. Filtering using all 25 possible combinations of these starting parameter values yielded a 5 × 5 matrix of resulting correlations (Supplementary Figure S3). The parameter values yielding the highest correlation were then selected to be the central points in the subsequently tested value ranges of these parameters. For example, among the parameters used above, if the highest correlation was found by using the *maximal coverage distance* of 300 and *minimal A-SNR length* of 4, the subsequently tested values were:

*Maximal coverage distance*:   100, 200, 300, 400, 500

*Minimal A-SNR length*:     0, 2, 4, 6, 8

This process was repeated until the maximal resolution was achieved and no further iterations were possible. Such optimization was carried out for sequential values of *the maximal number of mismatches* starting with zero until no further improvement in correlation was observed.

### Ranking genes by likelihood to attract internal priming

Optimally filtered 10X PBMC1 dataset by Ding *et al.* (22) was used to rank the genes. We used R package zoib (28) to derive the per-gene dependence of proportion of optimally filtered out alignments on the genes’ exonic length and A-SNR content, using a Bayesian zero-one inflated beta regression model, as follows:

$$\begin{equation*} E(Y) = \beta _{0} + \beta _{1}L + \beta _{2}S_{1} + \beta _{3}S_{2} + \beta _{4}S_{3} + ... + \beta _{50}S_{55} \end{equation*}$$

where *E*(*Y*) ∈ [0, 1] is the expected proportion of internally primed reads removed by filtering, *L* is the collapsed exonic length of the gene in bp, and *S*_*N*_ is the number of exonic A-SNRs of length *N* (including up to 1 mismatch) contained in the given gene, as per the GRCh38 v1.2.0 Cell Ranger human reference genome (used by the authors of this dataset). Note that not all A-SNR lengths below 55 were represented in the model due to their absence among exonic A-SNRs across the expressed genes used to derive this model. The specific model used was selected as the one that generated the lowest mean squared error (MSE) on out-of-sample data among all tested models. The mean of the Bayesian posterior predictive sample (*n* = 800) was used as the resulting prediction, *E*(*Y*), for each gene. All human genes were subsequently rank-ordered by these predictions. The posterior predictive sample for Gene ‘GREM1’ (ID ENSG00000166923) yielded 751 ‘NA’ values due to their calculation involving numbers too large for computation using R but whose value is expected to be approaching 1. The prediction for this gene, which was made based on the remaining values (*n* = 49) that were all very close or equal to 1, is therefore expected to be accurately reflective of or slightly lower than the true prediction for this gene, having no effect on it already being the top-ranked gene.

### Code availability

The python code for the BAM filtering algorithm as well as all other analyses described in this publication is available at https://github.com/MarekSvob/polyAfilter.

## RESULTS

### External RNA Controls Consortium (ERCC) spike-in standards exhibit signs of internal oligo(dT) priming

ERCC standards are synthetic RNA molecules with poly(A) tails that are routinely used for baseline measurements and normalization of RNA sequencing data by comparing the known input ERCC concentrations to the sequenced output quantities (29). To assess whether oligo(dT)s may be prone to internal priming, we first set out to examine a publicly available dataset by Wu *et al.* (20), which was enriched with ERCC spike-in standards. This dataset was generated using the Lexogen QuantSeq 3′ mRNA-Seq REV (‘Lexo REV’) library prep kit, which is a bulk RNA sequencing protocol that aims to pinpoint the exact 3′ ends of transcripts by generating antisense reads directly upstream of the oligo(dT) priming event (Figure 1A). We reasoned that if internal priming did occur during the creation of this dataset, we would be able to detect outliers among the ERCC standards whose measured output alignment counts are higher than expected from their input concentrations.

Indeed, by comparing the known input concentrations of the ERCC standards to their respective resulting alignment counts after sequencing, we detected several notable outliers in the data (Figure 2A). After removal of all non-terminal ERCC alignments, which mapped further (i.e. more than 75 base pairs (bp)) upstream than reasonably expected to result from the oligo(dT)s priming onto the poly(A) tails, the outliers’ alignment counts moved closer to their expected values and the overall correlation of the input concentrations with the detected output counts increased significantly (Pearson’s r increased from 0.91 to 0.94, *P* ≈ 0.006 calculated using two-tailed Williams’ *t*-test (30); Figure 2B). We examined where the alignments mapped on the ERCC reference sequences and found that a large portion of the ERCC-associated non-terminal alignments mapped directly upstream of internal SNRs of five or more consecutive As (Figure 2C). ERCC spike-in standards therefore may be subject to internal oligo(dT) priming, possibly secondary to oligo(dT)s’ hybridization to A-rich internal sequences on the ERCC standards. Interestingly, Wu *et al.* did not use the ERCC sequencing data in their final data analyses, citing inconsistent results.

### Oligo(dT)-primed RNA sequencing reads are enriched upstream of genome encoded poly(A) sequences

To determine if internal priming might also be detected in experimental RNA sequencing data, we first scanned the human genome for the presence of A-SNRs on both strands (i.e. A-SNRs and T-SNRs, which are equivalent to A-SNRs found on the opposite strand of the genome) and quantified their relative abundance by length (Figure 3A; for scanning results of other commonly used reference genomes, see Supplementary Figure S1). As previously reported for both A/T- and C/G-SNRs (27), the relative frequency of A-SNRs nine or more nucleotides long normalized by their expected frequency increased logarithmically with increasing A-SNR length, suggesting the presence of evolutionary processes that favor long stretches of adenines and hence provide more opportunities for internal oligo(dT) priming. Since most RNA sequencing gene expression data analyses commonly focus on reads that specifically align to exonic sequences of genes, we also determined the relative distribution of A-SNRs with respect to their genomic annotations (Figure 3B and Supplementary Figure S1). We found that while the abundance of A-SNRs was relatively depleted in exons compared to other genomic annotations, exons still contained a considerable proportion of A-SNRs, rendering these sites viable candidates for internal oligo(dT) priming that could potentially result in inflated exonic sequencing read counts.

We, therefore, measured aggregate exonic RNA sequencing coverage upstream of potential priming events in three datasets, each created using a different library preparation method but all utilizing oligo(dT) priming: the Lexo REV dataset by Wu *et al.* discussed above (bulk RNA sequencing in the antisense orientation) (20), a Lexogen QuantSeq 3′ mRNA-Seq FWD dataset by Ma *et al.* (‘Lexo FWD,’ bulk RNA sequencing in the sense orientation) (21), and a 10X Genomics Chromium Single Cell 3′ (v2) dataset by Ding *et al.* (‘10X’, scRNAseq in the sense orientation) (22). First, we measured aggregate exonic RNA sequencing coverage aligned in the vicinity of annotated transcript 3′ ends (terminal coverage) across the respective reference genomes. As expected, we observed an enrichment of sequencing reads directly upstream (Figure 3C) of transcript 3′ ends, originating from terminal oligo(dT) priming onto poly(A) tails of mRNA molecules. Also as expected from the differences between the respective library preparation methods (Figure 1A), alignment enrichment in the Lexo REV dataset was found directly upstream of and adjacent to the transcript 3′ ends (reads being in the antisense orientation with respect to the transcript reference), while the enrichment peaks were further upstream in the Lexo FWD and 10X datasets (with reads in the sense orientation). Analogously, we scanned the same datasets for aggregate exonic coverage in the vicinity of exonic SNRs of five or more continuous adenines (internal coverage) across the respective genomes and intriguingly, we also found enrichment directly upstream of these loci and the distances of the enrichment peaks from the A-SNRs mirrored those seen upstream of transcript 3′ ends, as described above (Figure 3D). The most likely explanation for this internal coverage enrichment is that these sequencing reads resulted from oligo(dT)s priming onto the A-SNRs found in the sequenced mRNA molecules. Moreover, the relative enrichment upstream of A-SNRs increased with increasing A-SNR length, suggesting that longer stretches of A-SNRs are more likely to attract oligo(dT) priming (Supplementary Figure S2).

### Removal of internally primed reads improves the accuracy of oligo(dT)-based RNA sequencing data

We next sought to investigate whether removal of internal alignments upstream of A-SNRs may lead to improved accuracy of mRNA counts in oligo(dT)-primed RNA sequencing data. For this purpose, we focused on the analysis of publicly available datasets that had been generated using the most common bulk and single-cell RNA sequencing library preparation methods for counting of mRNA molecules via oligo(dT) priming and sense-oriented sequencing (Lexo FWD bulk dataset by Ma *et al.* (21), and 10X, CEL-seq2, Drop-Seq, inDrop and Seq-well single-cell datasets by Ding *et al.* (22)). Each of these datasets also had an associated random oligo-primed bulk RNA sequencing dataset generated from the same biological sample used as a gold standard to correlate their gene expression measurement accuracy.

Due to the constraints of next-generation sequencing, all of these methods are optimized to yield cDNA libraries with fragments of a length limited to about 500 bp (e.g. the mean fragment size including adaptors for the QuantSeq libraries is 335–456 bp, with the cDNA insert size being 203–324 bp; https://faqs.lexogen.com/faq/What-is-the-typical-fragment-size-for-QuantSeq-Libraries/3F.118686112.html). In these oligo(dT) priming-based methods, the reads that align to the reference genome are sequenced from the 5′ end toward the 3′ end in the sense strand (FWD) orientation and are usually less than 100 bp long (Figure 1A). As a result, these sequencing reads align to the reference genome up to several hundred bps upstream of the region corresponding to the original oligo(dT) priming event (either onto a poly(A) tail or an A-SNR), complicating the association of the priming event with the resulting sequencing read alignment.

Notably, this distance gap between the priming event and the associated sequencing read alignment is observed in the FWD sequencing methods, which focus on mRNA counting. Methods that instead focus on polyadenylation site detection commonly use sequencing reads from the 3′ end toward the 5′ end of the mRNA in the antisense strand (REV) orientation directly upstream of and adjacent to the oligo(dT) priming event (i.e. with no distance gap, Figure 1A), which is used in pinpointing the exact site of polyadenylation in these studies. This difference in the presence of the gap is also illustrated in Figure 3C, D, where the peak sequencing coverage from the ‘Lexo REV’ dataset is always directly adjacent to the respective priming events, unlike the peaks from the ‘Lexo FWD’ and ‘10X’ datasets, which are located more upstream. Moreover, in the REV sequencing data, the terminal and internal oligo(dT) priming events can be distinguished due to the fact that the terminal alignments contain the polyadenylation signal and the internally primed alignments show adenine enrichment (A-SNRs) directly downstream.

However, because the sequencing read alignments in the RNA counting FWD library preparation methods primarily discussed here are not adjacent to their oligo(dT) priming sites, terminally and internally primed alignments cannot be distinguished based on the presence of polyadenylation signals in the reads or adenine enrichment directly downstream and, therefore, filtering methods previously applied in polyadenylation studies using REV sequencing cannot be applied in gene counting methods that use FWD sequencing. Instead, a completely novel filtering algorithm has to be developed to take into account the distance (i.e. the *maximal coverage distance*) of the alignments from their respective priming events (3′ transcript ends for terminal priming and A-SNRs for internal priming) to correctly identify and remove the sequencing read alignments that likely resulted from internal priming (Figure 1B).

In addition to the *maximal coverage distance*, the minimal length of an A-SNR that is sufficient for oligo(dT) priming to occur (*minimal A-SNR length*) also has to be determined for correct identification of internally primed alignments. Finally, as we suspected that A-SNRs may attract oligo(dT) priming even if they contain one or several other bases, we also had to determine the *maximal number of mismatches* allowed in A-SNRs for internal priming to occur in these library preparation methods.

**Figure 4.**
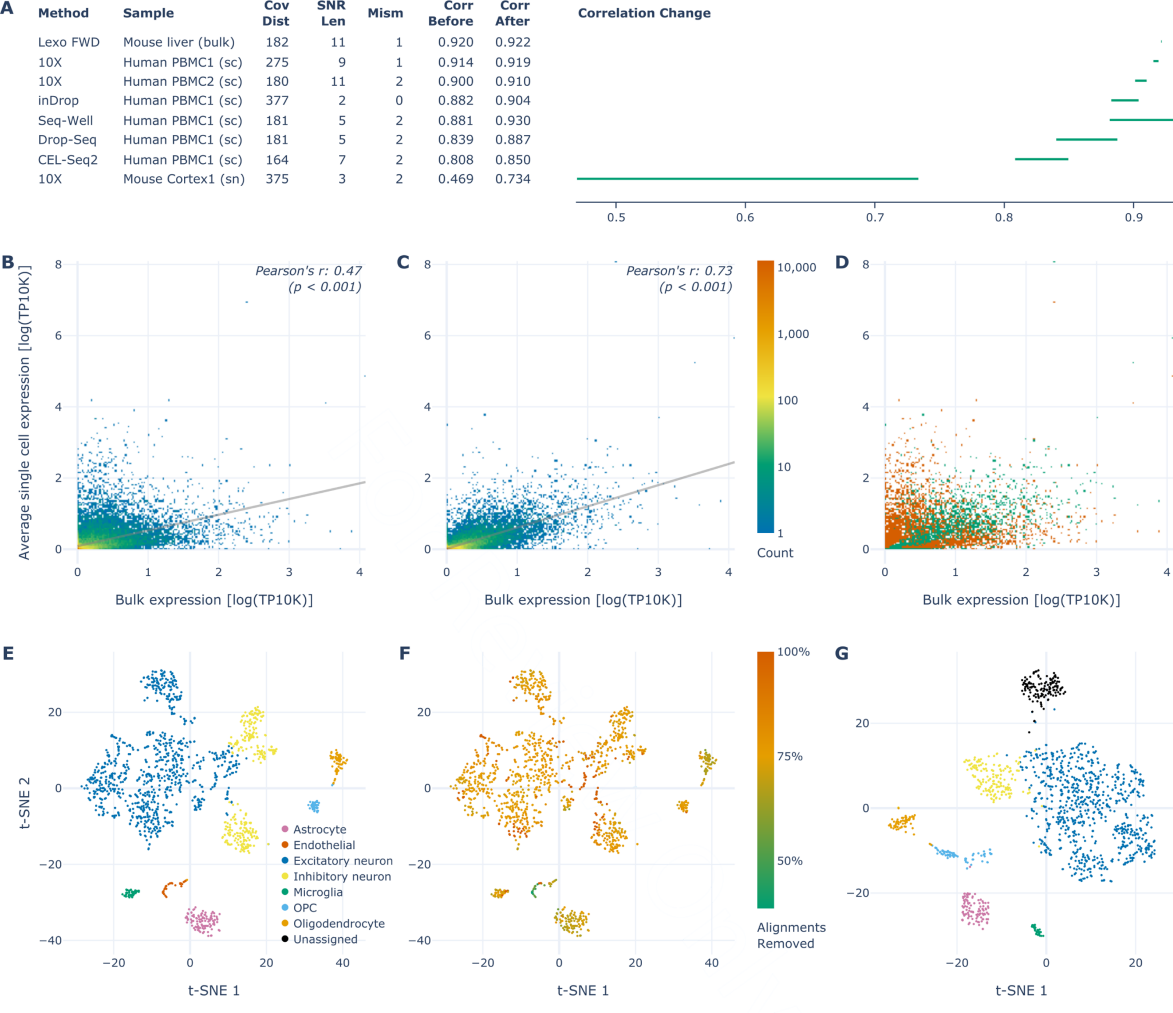
Optimized filtering parameters by library preparation method and details of the single nuclei dataset. (**A**) Table of the optimized filtering values for each respective dataset studied along with the correlation change (visualized difference between the correlation before and after filtering; single-cell correlation values before filtering reproduced from Supplementary Figure 11 in (22)). *P* << 0.001 for each correlation, as well as for each difference between the correlations before and after filtering (adjusted using the Bonferroni correction). Abbreviations used: ‘Cov Dist’: maximal coverage distance; ‘SNR Len’: minimal A-SNR length; ‘Mism’: maximal number of mismatches; ‘Corr’: correlation; ‘sc’: single cells; ‘sn’: single nuclei. (**B**) Correlation of gene expression in the sample of mouse cortex nuclei between the 10X (oligo(dT)) and bulk (random oligos) methods, with intronic alignments included in gene expression quantification for both. (**C**) Correlation between the same datasets as in (B) after filtering the oligo(dT) dataset using the optimal parameters for this dataset listed in (A). Both (B) and (C) are 2D histograms sharing the rainbow color scale depicting the density of genes in each region with the line of best fit in gray. (**D**) Visualization of changes between (B) and (C), where the regions with fewer and more genes in (C) relative to (B) are depicted by orange and green, respectively. (**E**) A t-SNE plot of the single nuclei mouse cortex 10X dataset colored by the cell types detected (reproduced from Figure 6A in (22)). (**F**) The same t-SNE plot as in (E), colored by the proportion of alignments optimally filtered out from each cell, as per the adjacent color scale. (**G**) A t-SNE plot of the same dataset as in (E) and (F), after the internally primed alignments have been optimally filtered out. The colors correspond to the same cell types as in (E). (*P*-values were calculated using a two-tailed *t*-test.)

We, therefore, developed an algorithm that filters internally primed sequencing reads from an aligned RNA sequencing dataset given the parameters of *maximal coverage distance*, *minimal A-SNR length* and the *maximal number of mismatches* allowed (Figure 1B). By iteratively applying this filtering algorithm, we then optimized these three parameters separately for each oligo(dT) priming-based dataset studied to maximize its accuracy (Supplementary Figure S3), i.e. its correlation with a gold standard bulk RNA sequencing dataset (one that uses random oligo priming) obtained from the same biological sample. We found that the optimal parameters, as well as the relative increase in correlation, differed across the methods but for each of the datasets studied, such a combination of filtering parameters could be found that resulted in a statistically significant increase in RNA sequencing accuracy (Figure 4A). Notably, although removal of all non-terminal alignments (i.e. those outside the *maximal coverage distance* upstream of transcript 3′ ends, regardless of their association with A-SNRs) improved the data accuracy in all non-bulk datasets for at least some values of *maximal coverage distance*, in all the datasets studied the highest increase in accuracy was observed after specific removal of only those alignments that were associated with A-SNRs. Additionally, there was no correlation between the number of exonic alignments removed and the resulting accuracy of the dataset (Supplementary Figure S4). When a proportion of exonic alignments equal to that in the optimally filtered dataset was removed randomly (i.e. regardless of their association with A-SNRs or transcript 3′ ends), the accuracy of the dataset decreased compared to the baseline before filtering (Supplementary Figure S4D). These results support the idea that internal priming events from A-SNRs specifically contribute to erroneous sequencing reads and thus decrease the accuracy of gene expression quantification.

The improvement of accuracy was the highest for the single nuclei dataset, where sequencing reads aligned to introns (along with those aligned to exons) were included in gene expression quantification (Figure 4B–G), as is common for single nuclei sequencing datasets (22,31). As expected from the higher relative abundance of A-SNRs in introns than in exons (Figure 3B), this dataset had the highest proportion of alignments filtered out, removing about 64% of intragenic (exonic and intronic) alignments overall (Supplementary Table S1), ranging from 38% to 100% per cell (Figure 4F). Interestingly, after filtering, the ‘Endothelial’ cell type was no longer detected in this mouse cortex sample, while one of the two cell clusters previously assigned to the ‘Inhibitory neuron’ cell type was now ‘Unassigned,’ confirming the spatial separation of these two cell clusters in the t-SNE plot as indicative of a difference in cell types (Figure 4G). When we analyzed this dataset considering only exonic alignments (as is common for single-cell datasets), the correlation with the associated bulk dataset was significantly higher than with intronic alignments included before filtering (0.710 without, compared to 0.469 with intronic alignments included) and it further increased after optimal filtering (0.753 compared to 0.734, respectively; Supplementary Figure S5). Inclusion (or lack thereof) of intronic alignments in the analysis of each associated random oligo bulk dataset mirrored that of the oligo(dT) dataset it was compared to. Because introns contain a relatively high abundance of A-SNRs, which are associated with spurious sequencing reads, the inclusion of intronic alignments likely further decreases the accuracy of gene expression quantification.

### A linear model ranks genes by the likelihood that their reads result from internal oligo(dT) priming

Of the methods studied, 10X is currently the most widely used mRNA counting library preparation method, as it is the most user-friendly and it shows the most consistency in results among the single-cell methods (22). Additionally, its accuracy was the highest at baseline before the filtering, and also the *minimal A-SNR length* for optimal filtering was the highest among the single-cell methods tested (Figure 4A), resulting in the lowest proportion of alignments removed (Supplementary Table S1). Therefore, we used the 10X Human PBMC1 single-cell dataset by Ding *et al.* (22) to derive a conservative linear model and predict the proportion of sequencing reads optimally filtered out for each human gene as a function of its exonic length and number of A-SNRs it contains. Using these predictions, we ranked all genes by the likelihood that their alignments result from internal oligo(dT) priming (Supplementary Figure S6 and Supplementary Table S2; the extended version of this table is available online: https://svoboda.shinyapps.io/SNRtable/). These results suggest that in oligo(dT) priming-based sequencing count methods, internal priming accounts for at least 10% of measured expression in over half of the genes and for over 1.4% of the genes, internal oligo(dT) priming may represent >50% of the measured expression, highlighting the uneven distribution of this effect among the genes and therefore constituting a systematic bias.

**Figure 5.**
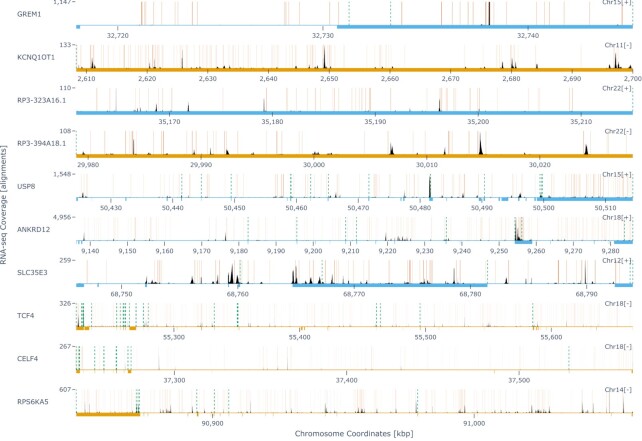
Sequencing coverage of the top 10 genes predicted to be affected by internal oligo(dT) priming. Genome tracks of each respective gene with the chromosome coordinates (x-axes) shown in the ‘+’ strand orientation. The sense strand of each gene is indicated next to the chromosome number (‘Chr#[+/-]’) in the upper right corner of each genome track and in the color of the track with light blue signifying the ‘+’ strand (displayed in 5′→3′ orientation) and light orange color signifying the ‘-’ strand (displayed in 3′→5′ orientation). The thick bars underneath the genome tracks depict the extent of exon annotations collapsed across all transcripts for each respective gene. Per-basepair sequencing coverage is shown in black with the maximum coverage indicated on the y-axis. The locations of genome-encoded SNRs of nine or more adenines with up to one mismatch are indicated by the solid orange vertical lines. The different 3′ ends (polyadenylation sites) for each mRNA transcript variant are indicated by the dashed green vertical lines. In the absence of internal poly(dT) priming, sequencing coverage would be expected to be detected only upstream (in each gene’s sense orientation) of these 3′ ends.

As seen in Figure 5, the ten genes with the highest predicted probabilities of internal oligo(dT) priming all contain numerous A-SNRs of lengths nine or higher with up to one mismatch, as per the optimal filtering parameters for this dataset (Figure 4A). All of these genes were detected as expressed in the 10X Human PBMC1 single-cell dataset, with only a minority of the sequencing reads aligned in the terminal portions of annotated transcripts (i.e. upstream of the transcript 3′ ends), while most of the alignment peaks were found upstream of the genome-encoded A-SNRs mentioned above. This observation strongly suggests that these alignments originated from internal oligo(dT) priming. Although these data represent sequencing of single-cell RNA (as opposed to that from single nuclei, which is expected to contain a higher proportion of intronic sequences) and the gene predictions were made with respect to only exonic A-SNRs, the top ten genes show a considerable amount of intronic coverage as well, in agreement with previous studies (18). Interestingly, cells expressing the second-highest ranked gene, KCNQ1OT1, have been previously discarded from oligo(dT) priming-based scRNA-seq datasets due to the ‘artifactual’ expression of this gene (32). Filtering out internally primed alignments may have obviated the need to discard cells from these scRNA-seq datasets.

## DISCUSSION

Oligo(dT) priming-based sequencing has revolutionized biology in recent years by enabling massively parallel sequencing of RNA from individual cells, while also inspiring the emergence of more economical bulk RNA sequencing methods. As next-generation sequencing is being gradually replaced by third-generation sequencing, which produces much longer reads without the need for sequence amplification (33), oligo(dT) priming-based methods have the distinct advantage of dispensing with the need to sequence the entire RNA molecule, instead focusing solely on mRNA counting to quantify gene expression. In this study, using publicly accessible datasets, we uncover a heretofore unexplored source of systematic bias in the oligo(dT)-primed forward RNA sequencing count data, whereby the underlying assumption that the number of cDNA fragments is proportional to the number of mRNA molecules does not hold true for all genes.

Using a dataset with ERCC spike-in control RNA fragments, we show that when non-terminal alignments are removed, the resulting accuracy of the output measurement of known input quantities improves significantly. In the subsequent analysis, we specifically pinpoint internal, genome-encoded A-SNRs as the most likely culprit by showing that there is a significant RNA coverage enrichment upstream of these sequences and that this enrichment increases with the increasing length of these A-SNRs. Based on these findings, we then developed the first algorithm to remove sequencing alignments that likely originated from internal oligo(dT) priming in forward RNA sequencing count methods. We show that in all datasets studied for which a gold standard measurement is available, removal of sequencing read alignments upstream of A-SNRs significantly improves the data accuracy. We, therefore, infer that the phenomenon of internal priming is most likely common to all oligo(dT) priming-based RNA counting approaches, which includes virtually all currently used scRNA-seq methods.

Additionally, in all datasets tested, the resulting accuracy after such specific optimized filtering was better than after indiscriminate removal of all non-terminal alignments (i.e. the optimal *minimal A-SNR length* was always higher than zero, see Materials and Methods). This is probably the result of the genome reference transcript annotations to date not yet being fully comprehensive. Because of the still incomplete knowledge of alternative polyadenylation sites across the genome, some of the alignments that are labeled non-terminal may in fact be upstream of an unknown biologically relevant transcript 3′ end and their indiscriminate removal, which likely occurs to a much lesser extent during the specific removal of alignments upstream of A-SNRs, can lead to suboptimal data accuracy.

From our iterative optimization, we found that the optimal filtering parameters (*maximal coverage distance*, *minimal A-SNR length* and *the maximal number of mismatches*) differ between datasets, which could be caused by the protocol-specific differences. Outside of this study, these parameters may further vary across different tissue types, research groups, and batches. As the optimal filtering parameters may be dataset-specific, we propose that obtaining a random oligo-primed measurement from the same sample for filtering parameter optimization may be the best way to ensure maximal possible accuracy of oligo(dT)-primed data.

However, most oligo(dT)-primed datasets usually do not have their random oligo-primed counterparts to provide a gold standard measurement. Additionally, the filtering parameter optimization process for each dataset is relatively time-consuming, even with the use of high-performance computing. Depending on the size of the dataset, a single filtering task required up to about three hours of CPU time (running a parallel process across 16 CPUs and using up to 120GB of RAM) and in order to find the optimal parameters for a given dataset, it had to be filtered about 300 times, using a different set of filtering parameters each time to find their optimal combination. Depending on the computational resources available, the time required to carry out this parameter optimization process can be significantly reduced by running these filtering tasks in parallel and using randomly subsampled alignment files.

Therefore, alternatively, candidate values of these parameters may be estimated from the results in this study, ensuring that filtering improves the accuracy of the sequencing data, although perhaps not optimally so. Using an *a priori* known set of parameters, the filtering algorithm may only be run once, significantly decreasing the computational time required. In all datasets studied, almost all combinations of the three filtering parameters yielded improvement in data accuracy. One notable exception was the Lexo FWD bulk dataset where filtering using values of *minimal A-SNR length* less than nine sometimes yielded a decrease in accuracy, whereby decreasing the *maximal number of mismatches* allowed for lower *minimal A-SNR lengths* to be still beneficial to the filtering process. Similarly, in both 10X single-cell datasets studied, only combinations of low *maximal coverage distance* and low *minimal A-SNR length* yielded a decrease in data accuracy, especially at the higher *maximal number of mismatches* allowed. In general, however, our observations suggest that filtering using our algorithm with the *maximal coverage distance* of 300 and the *minimal A-SNR length* of 10 with up to one *mismatch* should be guaranteed to improve the accuracy of any oligo(dT) priming-based dataset.

Future studies may be needed to provide additional clarifications about the nature of this phenomenon and further optimize the data filtration algorithm. For example, in this study, we assumed that internal oligo(dT) priming is most likely to occur on genome-encoded poly(A) sequences with only occasional (up to three) mismatches. While it is well established that thymine nucleotides best hybridize to adenines, their selective preference for the three possible ‘mismatches’ is less clear. For example, a previous study suggests that oligo(dT)s may have a high hybridization rate also onto AG-rich sequences (15). Furthermore, we did not focus on RNA sequencing methods that, following oligo(dT) priming, use full transcript length sequencing to provide gene expression quantification as well as splice variant information, such as SMART-seq. We hypothesize that these methods may be similarly affected by internal priming, although to a different extent.

Nevertheless, the findings in this study clearly demonstrate that caution should be exercised when using oligo(dT) priming-based forward RNA sequencing library preparation methods. An additional step of data analysis in the form of filtering of the alignments that likely resulted from internal oligo(dT) priming should be carried out, especially when directly comparing relative expression of one gene to another or inferring new 3′ untranslated region annotations from this data, with special attention given to the genes we have highlighted as those most likely to be affected by internal priming.

## Supplementary Material

lqac035_Supplemental_File

## References

[1] Eberwine J. , Yeh H., Miyashiro K., Cao Y., Nair S., Finnell R., Zettel M., Coleman P. Analysis of gene expression in single live neurons. Proc. Nat. Acad. Sci. U.S.A. 1992; 89:3010–3014. 10.1073/pnas.89.7.3010 PMC48793 1557406

[2] Macosko E.Z. , Basu A., Satija R., Nemesh J., Shekhar K., Goldman M., Tirosh I., Bialas A.R., Kamitaki N., Martersteck E.M. et al. Highly parallel genome-wide expression profiling of individual cells using nanoliter droplets. Cell. 2015; 161:1202–1214. 26000488 10.1016/j.cell.2015.05.002 PMC4481139

[3] Klein A. , Mazutis L., Akartuna I., Tallapragada N., Veres A., Li V., Peshkin L., Weitz D., Kirschner M. Droplet barcoding for single-cell transcriptomics applied to embryonic stem cells. Cell. 2015; 161:1187–1201. 26000487 10.1016/j.cell.2015.04.044 PMC4441768

[4] Zheng G.X. , Terry J.M., Belgrader P., Ryvkin P., Bent Z.W., Wilson R., Ziraldo S.B., Wheeler T.D., McDermott G.P., Zhu J. et al. Massively parallel digital transcriptional profiling of single cells. Nat. Commun. 2017; 8:14049. 28091601 10.1038/ncomms14049 PMC5241818

[5] Gierahn T.M. , Wadsworth M.H., Hughes T.K., Bryson B.D., Butler A., Satija R., Fortune S., Love J.C., Shalek A.K. Seq-Well: portable, low-cost RNA sequencing of single cells at high throughput. Nat. Methods. 2017; 14:395–398. 28192419 10.1038/nmeth.4179 PMC5376227

[6] Cao J. , Packer J.S., Ramani V., Cusanovich D.A., Huynh C., Daza R., Qiu X., Lee C., Furlan S.N., Steemers F.J. et al. Comprehensive single-cell transcriptional profiling of a multicellular organism. Science (New York, N.Y.). 2017; 357:661–667. 10.1126/science.aam8940 PMC5894354 28818938

[7] Hagemann-Jensen M. , Ziegenhain C., Chen P., Ramsköld D., Hendriks G.J., Larsson A.J., Faridani O.R., Sandberg R. Single-cell RNA counting at allele and isoform resolution using Smart-seq3. Nat. Biotechnol. 2020; 38:708–714. 32518404 10.1038/s41587-020-0497-0

[8] Hashimshony T. , Senderovich N., Avital G., Klochendler A., de Leeuw Y., Anavy L., Gennert D., Li S., Livak K.J., Rozenblatt-Rosen O. et al. CEL-Seq2: sensitive highly-multiplexed single-cell RNA-Seq. Genome Biol. 2016; 17:1–7. 27121950 10.1186/s13059-016-0938-8 PMC4848782

[9] Moll P. , Ante M., Seitz A., Reda T. QuantSeq 3′ mRNA sequencing for RNA quantification. Nat. Methods. 2014; 11:i–iii.

[10] Lohman B.K. , Weber J.N., Bolnick D.I. Evaluation of TagSeq, a reliable low-cost alternative for RNAseq. Mol. Ecol. Res. 2016; 16:1315–1321. 10.1111/1755-0998.12529 27037501

[11] Sholder G. , Lanz T.A., Moccia R., Quan J., Aparicio-Prat E., Stanton R., Xi H.S. 3′Pool-seq: an optimized cost-efficient and scalable method of whole-transcriptome gene expression profiling. BMC Genomics. 2020; 21:1–11. 10.1186/s12864-020-6478-3 PMC6971924 31959126

[12] Nam D.K. , Lee S., Zhou G., Cao X., Wang C., Clark T., Chen J., Rowley J.D., Wang S.M. Oligo(dT) primer generates a high frequency of truncated cDNAs through internal poly(A) priming during reverse transcription. Proc. Nat. Acad. Sci. U.S.A. 2002; 99:6152–6156. 10.1073/pnas.092140899 PMC122918 11972056

[13] Zhang H. , Hu J., Recce M., Tian B. PolyA_DB: A database for mammalian mRNA polyadenylation. Nucleic Acids Res. 2005; 33:D116–D120. 15608159 10.1093/nar/gki055 PMC540009

[14] Lee J.Y. , Yeh I., Park J.Y., Tian B. PolyA_DB 2: mRNA polyadenylation sites in vertebrate genes. Nucleic Acids Res. 2007; 35:D165–D168. 17202160 10.1093/nar/gkl870 PMC1899096

[15] Graber J.H. , Nazeer F.I., Yeh P.C., Kuehner J.N., Borikar S., Hoskinson D., Moore C.L. DNA damage induces targeted, genome-wide variation of poly(A) sites in budding yeast. Genome Res. 2013; 23:1690–1703. 23788651 10.1101/gr.144964.112 PMC3787265

[16] Wilkening S. , Pelechano V., Järvelin A.I., Tekkedil M.M., Anders S., Benes V., Steinmetz L.M. An efficient method for genome-wide polyadenylation site mapping and RNA quantification. Nucleic Acids Res. 2013; 41:e65. 23295673 10.1093/nar/gks1249 PMC3597643

[17] Roy K. , Gabunilas J., Gillespie A., Ngo D., Chanfreau G.F. Common genomic elements promote transcriptional and DNA replication roadblocks. Genome Res. 2016; 26:1363–1375. 27540088 10.1101/gr.204776.116 PMC5052057

[18] La Manno G. , Soldatov R., Zeisel A., Braun E., Hochgerner H., Petukhov V., Lidschreiber K., Kastriti M.E., Lönnerberg P., Furlan A. et al. RNA velocity of single cells. Nature. 2018; 560:494–498. 30089906 10.1038/s41586-018-0414-6 PMC6130801

[19] Vardi O. , Shamir I., Javasky E., Goren A., Simon I. Biases in the SMART-DNA library preparation method associated with genomic poly dA/dT sequences. PLoS One. 2017; 12:e0172769. 28235101 10.1371/journal.pone.0172769 PMC5325289

[20] Wu G. , Schmid M., Rib L., Polak P., Meola N., Sandelin A., Jensen T.H. A two-layered targeting mechanism underlies nuclear RNA sorting by the human exosome. Cell Rep. 2020; 30:2387–2401. 32075771 10.1016/j.celrep.2020.01.068

[21] Ma F. , Fuqua B.K., Hasin Y., Yukhtman C., Vulpe C.D., Lusis A.J., Pellegrini M. A comparison between whole transcript and 3′ RNA sequencing methods using Kapa and Lexogen library preparation methods. BMC Genomics. 2019; 20:9. 30616562 10.1186/s12864-018-5393-3 PMC6323698

[22] Ding J. , Adiconis X., Simmons S.K., Kowalczyk M.S., Hession C.C., Marjanovic N.D., Hughes T.K., Wadsworth M.H., Burks T., Nguyen L.T. et al. Systematic comparison of single-cell and single-nucleus RNA-sequencing methods. Nat. Biotechnol. 2020; 38:737–746. 32341560 10.1038/s41587-020-0465-8 PMC7289686

[23] Dobin A. , Davis C.A., Schlesinger F., Drenkow J., Zaleski C., Jha S., Batut P., Chaisson M., Gingeras T.R. STAR: ultrafast universal RNA-seq aligner. Bioinformatics. 2013; 29:15–21. 23104886 10.1093/bioinformatics/bts635 PMC3530905

[24] dos Santos G. , Schroeder A.J., Goodman J.L., Strelets V.B., Crosby M.A., Thurmond J., Emmert D.B., Gelbart W.M. FlyBase Consortium FlyBase: introduction of the *Drosophila melanogaster* release 6 reference genome assembly and large-scale migration of genome annotations. Nucleic Acids Res. 2015; 43:D690–D697. 25398896 10.1093/nar/gku1099 PMC4383921

[25] Eckardt N.A. Sequencing the rice genome. Plant Cell. 2000; 12:2011–2017. 11090205 10.1105/tpc.12.11.2011 PMC526008

[26] Cock P. J.A. , Antao T., Chang J.T., Chapman B.A., Cox C.J., Dalke A., Friedberg I., Hamelryck T., Kauff F., Wilczynski B. et al. Biopython: freely available Python tools for computational molecular biology and bioinformatics. Bioinformatics. 2009; 25:1422–1423. 19304878 10.1093/bioinformatics/btp163 PMC2682512

[27] Murray V. The frequency of poly(G) tracts in the human genome and their use as a sensor of DNA damage. Comput. Biol. Chem. 2015; 54:13–17. 25479163 10.1016/j.compbiolchem.2014.11.006

[28] Liu F. , Kong Y. zoib: An R package for Bayesian inference for beta regression and Zero/one inflated beta regression. R Journal. 2015; 7:34–51.

[29] Jiang L. , Schlesinger F., Davis C.A., Zhang Y., Li R., Salit M., Gingeras T.R., Oliver B. Synthetic spike-in standards for RNA-seq experiments. Genome Res. 2011; 21:1543–1551. 21816910 10.1101/gr.121095.111 PMC3166838

[30] Steiger J.H. Tests for comparing elements of a correlation matrix. Psychol. Bull. 1980; 87:245–251.

[31] Bakken T.E. , Hodge R.D., Miller J.A., Yao Z., Nguyen T.N., Aevermann B., Barkan E., Bertagnolli D., Casper T., Dee N. et al. Single-nucleus and single-cell transcriptomes compared in matched cortical cell types. PLoS One. 2018; 13:e0209648. 30586455 10.1371/journal.pone.0209648 PMC6306246

[32] Boisset J.-C. , Vivié J., Grün D., Muraro M.J., Lyubimova A., van Oudenaarden A. Mapping the physical network of cellular interactions. Nat. Methods. 2018; 15:547–553. 29786092 10.1038/s41592-018-0009-z

[33] Liu H. , Begik O., Lucas M.C., Ramirez J.M., Mason C.E., Wiener D., Schwartz S., Mattick J.S., Smith M.A., Novoa E.M. Accurate detection of m6A RNA modifications in native RNA sequences. Nat. Commun. 2019; 10:1–9. 31501426 10.1038/s41467-019-11713-9 PMC6734003

