# Supplementary material for: Internal oligo(dT) priming introduces systematic bias in bulk and single-cell RNA sequencing count data
Source: NAR Genom Bioinform. 2022 May 25;4(2):lqac035. doi: 10.1093/nargab/lqac035 (PMC9142200; doi:10.1093/nargab/lqac035)
Supplement: lqac035_Supplemental_File [file lqac035_supplemental_file.pdf]

SUPPLEMENTARY DATA

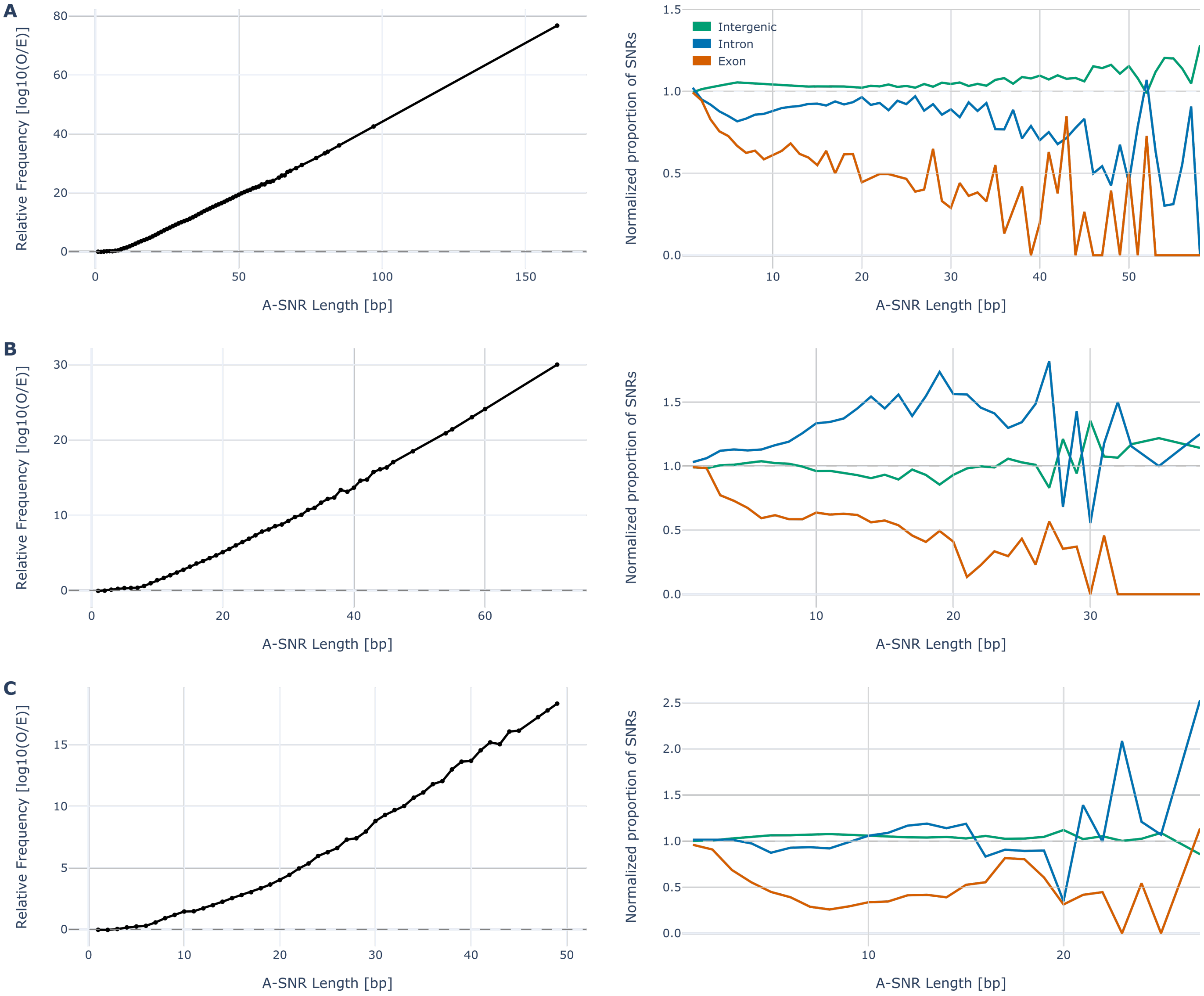

Figure S1. The relative frequencies of A-SNRs across various genome references. A-SNR analysis for reference genomes of (**A**) *Mus musculus*, (**B**) *Drosophila melanogaster*, and (**C**) *Oriza sativa*. Left: Log10 of relative (observed/expected) frequency of A-SNRs by length across the entire respective reference. The expected frequency was calculated based on the probability that A-SNRs of given length be found by pure chance. Right: The relative proportion of A-SNRs of each length by their genomic annotations, normalized by the respective proportions of the genome thus annotated. SNR lengths represented by fewer than 10 SNRs were removed. Color legend from (A) applies to (B) and (C) as well. Gray dashed line in each plot represents the expected value.

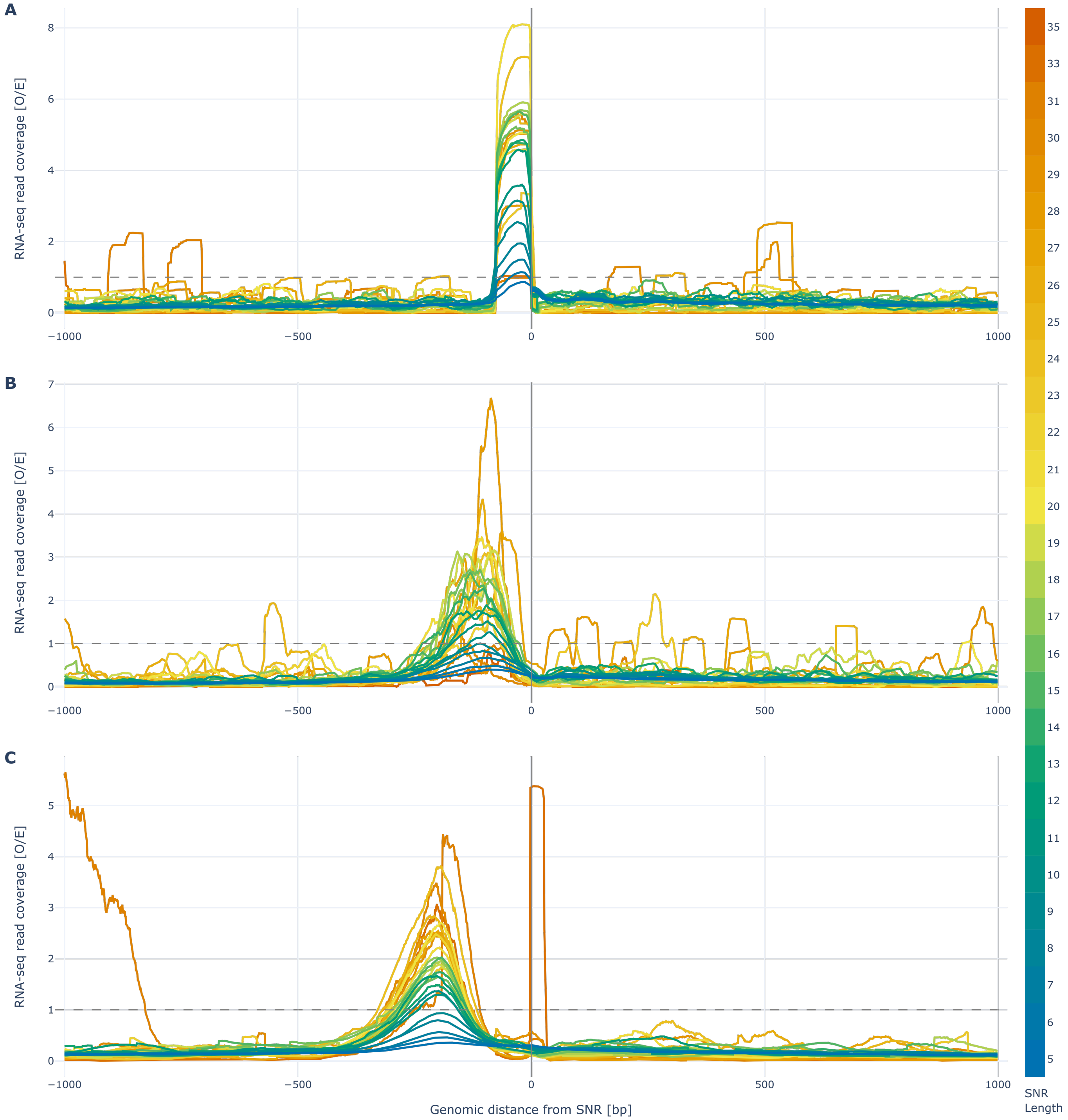

Figure S2. Sequencing coverage in the vicinity of SNRs by length. Normalized aggregate stranded exonic RNA sequencing coverage in the vicinity of A-SNRs of five nucleotides or longer, grouped by A-SNR length depicted by the color scale. The starts (5’ ends) of all A-SNRs are aligned at “0 bp” in the sense orientation. Sequencing coverage from the following datasets is depicted: (**A**) “Lexo REV” by Wu, Schmid, Rib *et al.* (1), (**B**) “Lexo FWD” by Ma *et al.* (2), and (**C**) “10X” by Ding *et al.* (3). In each graph, the gray dashed line represents the expected value if exonic coverage was randomly (evenly) distributed along exons. A-SNR outliers with associated coverage higher than 100× the expected value and A-SNR lengths represented by fewer than 10 A-SNRs were removed from this data.

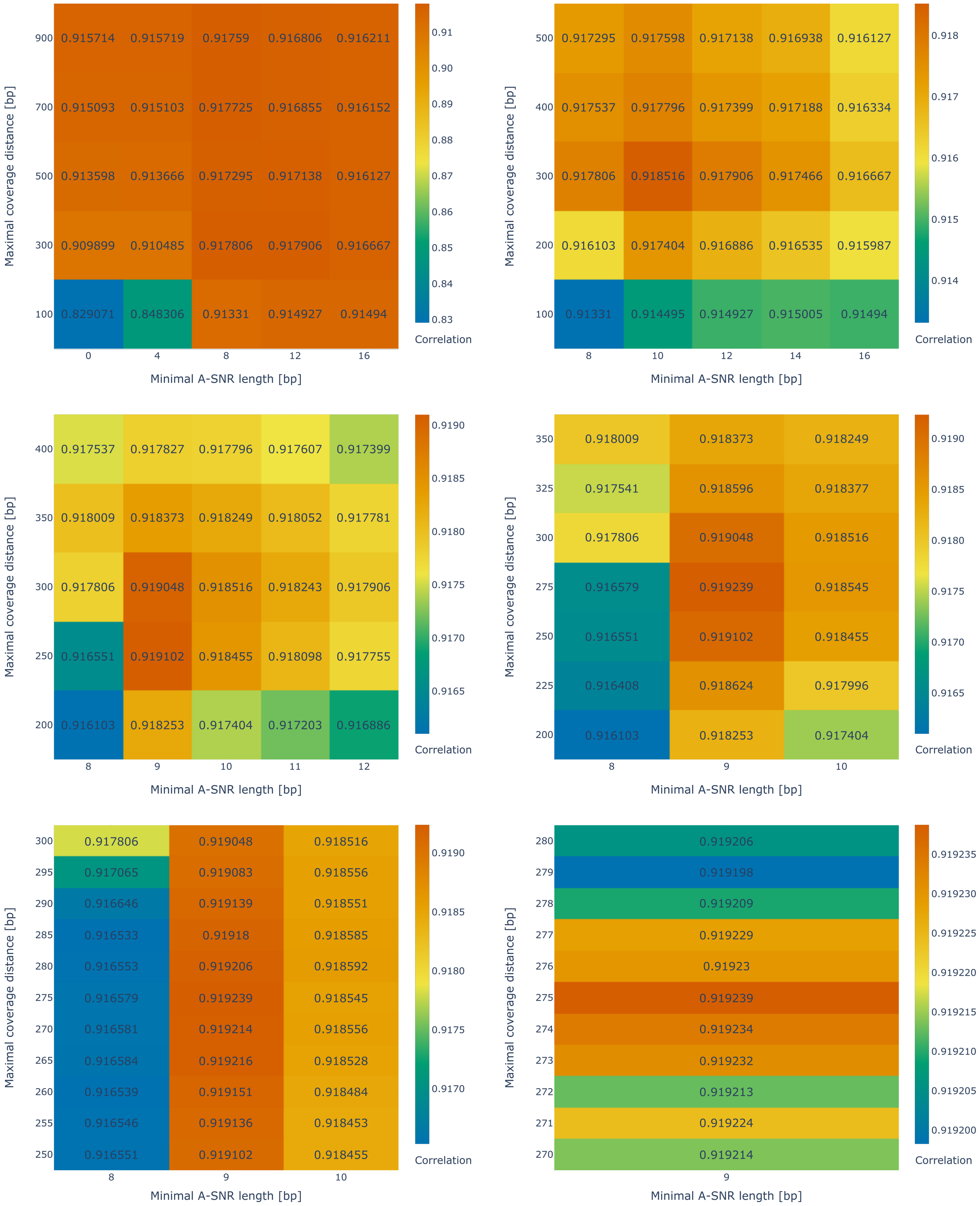

Figure S3. An example of parameter optimization through stepwise alignment filtering. Sequential steps in filtering optimization of the PBMC1 10X (v2) dataset by Ding *et al.* (3), at varying values of *minimal A-SNR length* and *maximal coverage distance*, with the *maximal number of mismatches* equal to one. Each subsequent set of parameters centers around those with the highest correlation(s) obtained at the previous step. Correlations with the associated bulk dataset in each heatmap are indicated by the number and color from the adjacent color scale.

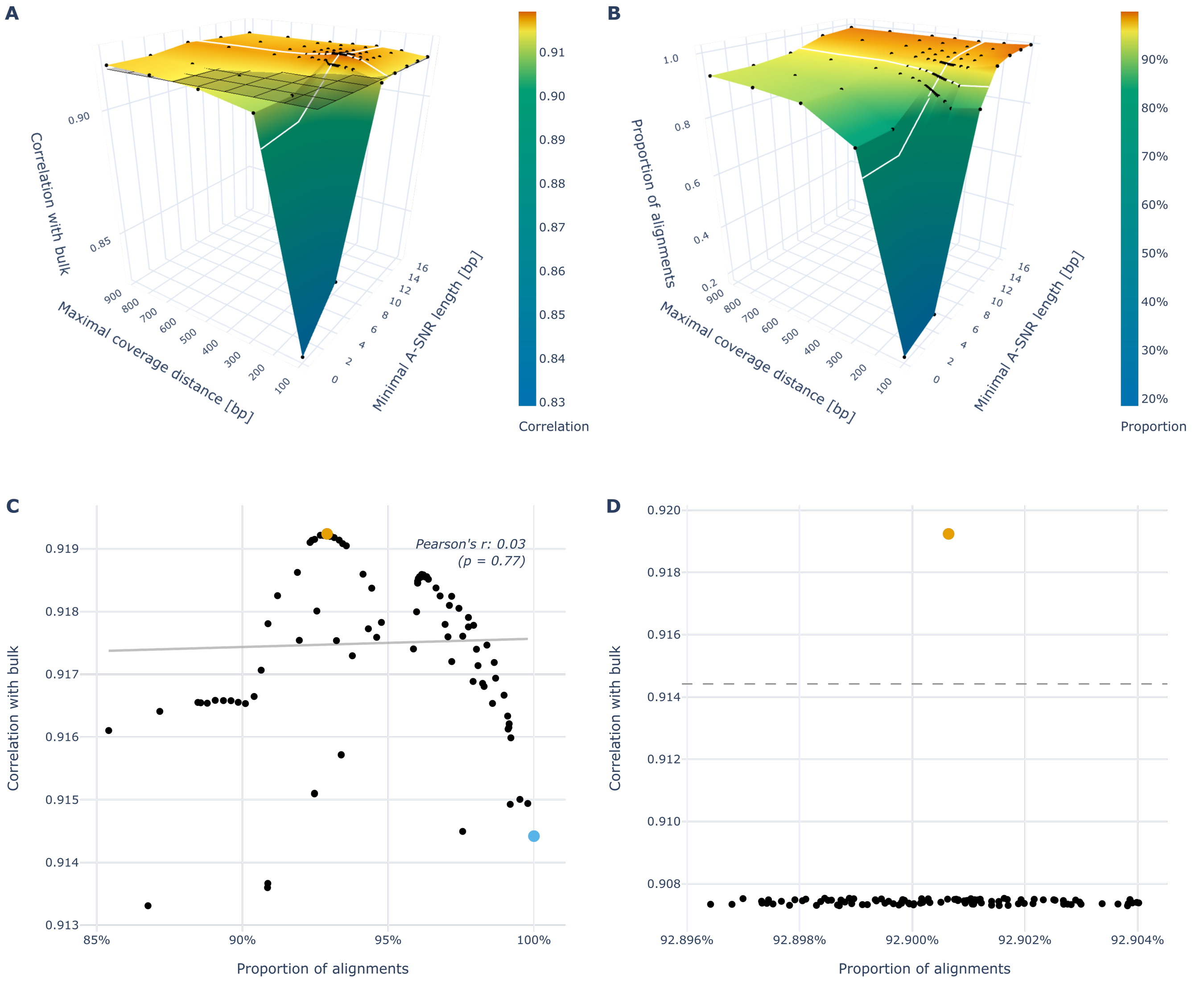

Figure S4. The correlation with bulk vs. the proportion of exonic alignments remaining after filtering. (**A**) A 3D surface representation of all the correlations from Supplementary Figure S3. The dark gray opaque plane with black orthogonal lines indicates the correlation of the original PBMC1 10X (v2) dataset by Ding *et al.* (3) with the associated bulk dataset before filtering. (**B**) A 3D surface representation of the remaining exonic alignment proportions after filtering for each respective combination of *minimal A-SNR length* and *maximal coverage distance* values from (A), with the value before filtering being equal to one (not shown). In both (A) and (B), the measured values are indicated as black dots with the surface interpolated in between and the white lines indicate the optimal *minimal A-SNR length* and *maximal coverage distance* whose combination yielded the highest correlation value after filtering. (**C**) A plot of correlations from (A) vs. proportions from (B) shows the lack of correlation (line of best fit in solid gray) between the two. Four outliers with proportions of exonic reads lower than 0.85 after filtering were removed. The blue dot represents the original dataset before filtering. (**D**) A plot of the remaining exonic alignment proportions vs. resulting correlations after 100 times randomly filtering out the same proportion of exonic alignments as in the optimally filtered dataset (∼7.1%; note that the slight variations in the final proportions are caused by varying the seed to initiate the pseudorandom filtering). The dashed gray line indicates the correlation before filtering. In both (C) and (D), the optimized dataset is represented by the orange dot. (*P*-value was calculated using two-tailed t-test.)

Method	Sample	Introns Included	Before Filtering		After Filtering	
			Exonic	Intronic	Exonic	Intronic
Lexo FWD	Mouse liver (bulk)	No	14 058 019	1 562 763	13 527 504 (96.2%)	1 562 763 (100.0%)
10X	Human PBMC1 (sc)	No	220 587 386	102 312 104	204 927 102 (92.9%)	102 312 104 (100.0%)
10X	Human PBMC2 (sc)	No	176 044 691	93 636 640	156 127 308 (88.7%)	93 636 640 (100.0%)
CEL-Seq2	Human PBMC1 (sc)	No	173 405 676	432 682 735	72 491 147 (41.8%)	432 682 735 (100.0%)
Drop-Seq	Human PBMC1 (sc)	No	155 733 230	62 564 219	70 809 314 (45.5%)	62 564 219 (100.0%)
inDrop	Human PBMC1 (sc)	No	165 292 779	104 599 750	131 549 685 (79.6%)	104 599 750 (100.0%)
Seq-Well	Human PBMC1 (sc)	No	70 258 471	48 897 396	24 399 738 (34.7%)	48 897 396 (100.0%)
10X	Mouse Cortex1 (sn)	Yes	121 416 773	133 732 777	89 931 124 (74.1%)	696 713 (0.5%)
10X	Mouse Cortex1 (sn)	No	121 416 773	133 732 777	93 787 604 (77.2%)	133 732 777 (100.0%)

Table S1. The numbers of intragenic alignments in each dataset’s BAM file before and after optimal filtering. The percentages indicate the proportion of the respective alignment types remaining after filtering.

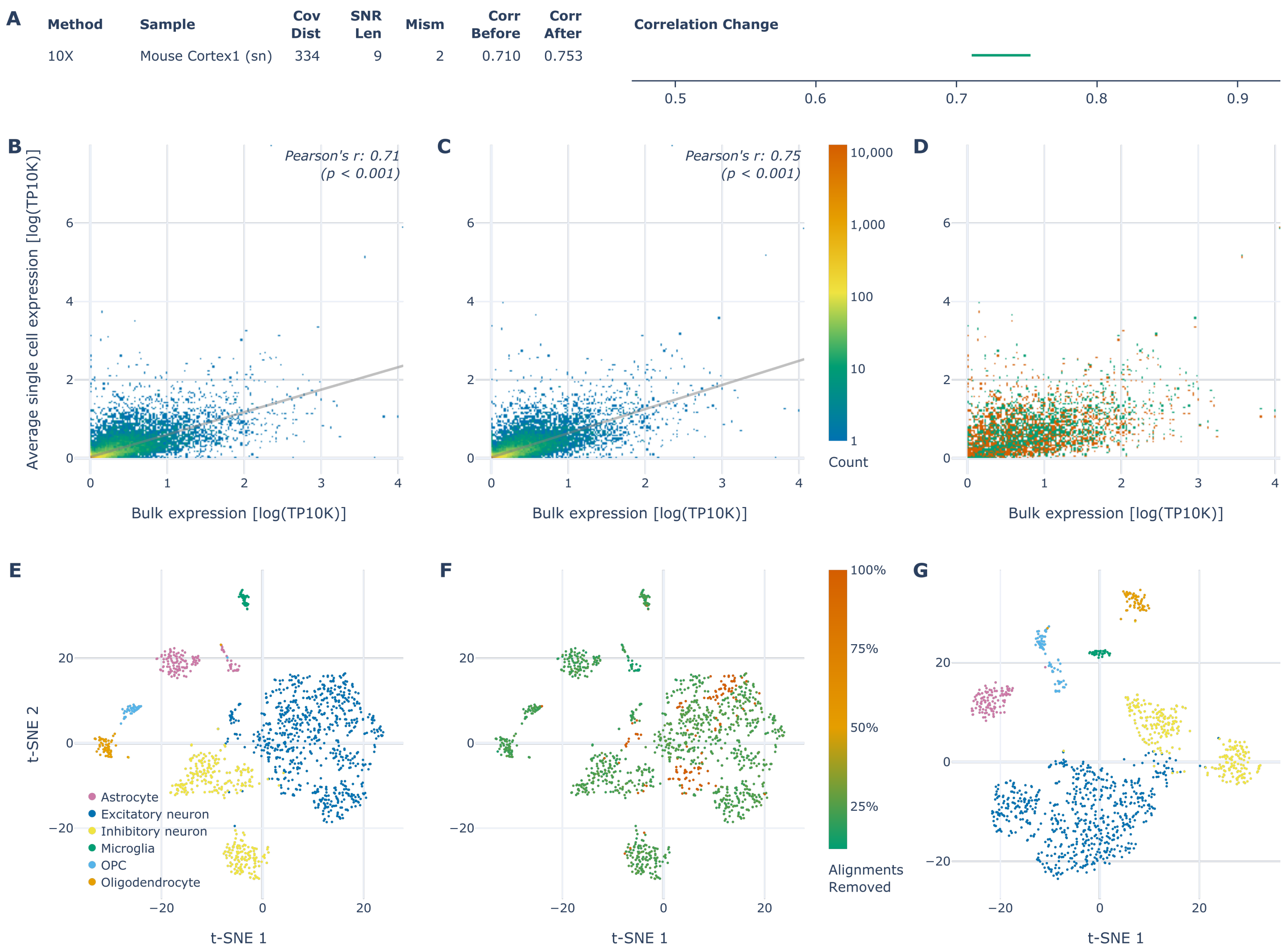

Figure S5. Optimized filtering of the single nuclei dataset with only exonic alignments included in gene expression quantification. (**A**) Table of the optimized filtering values for the Mouse Cortex1 single nuclei 10X dataset by Ding *et al.* (3) when intronic alignments are excluded from gene expression quantification (in both the single nuclei and the associated bulk datasets). Correlation change between before and after optimal filtering visualized on the same x-axis as in Figure 4A); $p << 0.001$ for each correlation, as well as for the difference between correlations before and after filtering, adjusted using the Bonferroni correction. Abbreviations used: “Cov Dist”: maximal coverage distance; “SNR Len”: minimal A-SNR length; “Mism”: maximal number of mismatches; “Corr”: correlation; “sn”: single nuclei. (**B**) Correlation of gene expression between the 10X (oligo(dT)) and bulk (random oligos) methods carried out on the same sample (mouse cortex single nuclei). (**C**) Correlation between the same datasets as in (B) after filtering the oligo(dT) dataset using the optimal parameters listed in (A). Both (B) and (C) are 2D histograms sharing the rainbow color scale depicting the density of genes in each region with the line of best fit in gray. (**D**) Visualization of changes between (B) and (C), where the regions with fewer and more genes in (C) relative to (B) are depicted by orange and green, respectively. (**E**) t-SNE plot of the single nuclei mouse cortex 10X dataset colored by the cell types detected. (**F**) The same t-SNE plot as in (E), colored by the proportion of alignments optimally filtered out from each cell, as per the adjacent color scale. (**G**) t-SNE plot of the same dataset as in (E) and (F), after the internally primed alignments have been optimally filtered out. The colors correspond to the same cell types as in (E). (P-values were calculated using two-tailed t-test.)

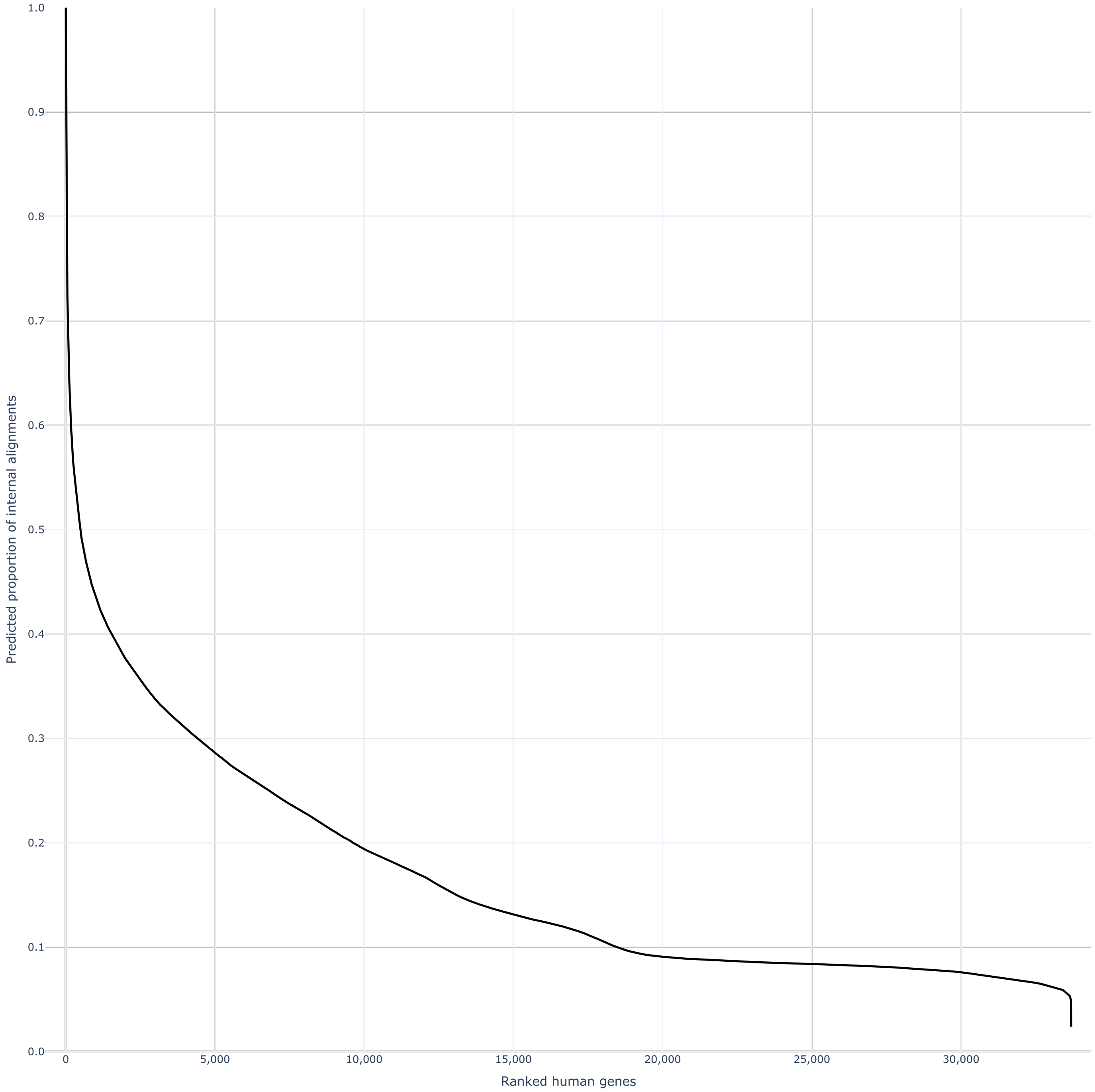

Figure S6. Ordered proportions of exonic alignments predicted by the linear model to originate from internal priming for all genes in the human genome when expressed.

Rank	Gene Name	Gene ID	Score
1	GREM1	ENSG00000166923	1.00
2	KCNQ1OT1	ENSG00000269821	1.00
3	RP3-323A16.1	ENSG00000279184	0.99
4	RP3-394A18.1	ENSG00000279159	0.96
5	USP8	ENSG00000138592	0.94
6	ANKRD12	ENSG00000101745	0.93
7	SLC35E3	ENSG00000175782	0.92
8	TCF4	ENSG00000196628	0.91
9	CELF4	ENSG00000101489	0.90
10	RPS6KA5	ENSG00000100784	0.88
11	TSIX	ENSG00000270641	0.88
12	PCDH9	ENSG00000184226	0.88
13	GABPB1-AS1	ENSG00000244879	0.86
14	CREBRF	ENSG00000164463	0.86
15	PCSK7	ENSG00000160613	0.85
16	NFIA	ENSG00000162599	0.85
17	SLC16A1-AS1	ENSG00000226419	0.84
18	RP4-671O14.7	ENSG00000280011	0.84
19	FLRT2	ENSG00000185070	0.84
20	CCDC168	ENSG00000175820	0.83
21	CYP20A1	ENSG00000119004	0.83
22	SRGAP1	ENSG00000196935	0.83
23	PSG2	ENSG00000242221	0.82
24	YIPF4	ENSG00000119820	0.82
25	CTA-228A9.4	ENSG00000279080	0.80
26	ZNF26	ENSG00000198393	0.80
27	TSHZ2	ENSG00000182463	0.80
28	LRTM1	ENSG00000144771	0.80
29	RP1-17K7.2	ENSG00000254584	0.80
30	CADM2	ENSG00000175161	0.80
31	AGO3	ENSG00000126070	0.79
32	PAAF1	ENSG00000175575	0.79
33	ZNF562	ENSG00000171466	0.79
34	IGF2BP2	ENSG00000073792	0.79
35	TNRC6B	ENSG00000100354	0.78
36	ELAVL3	ENSG00000196361	0.77
37	ONECUT2	ENSG00000119547	0.77
38	RBMS3	ENSG00000144642	0.77
39	BCYRN1	ENSG00000236824	0.77
40	MGAT4C	ENSG00000182050	0.77
41	LPP	ENSG00000145012	0.77
42	STK3	ENSG00000104375	0.77
43	KCNK6	ENSG00000099337	0.77
44	FAM133B	ENSG00000234545	0.76
45	CEP78	ENSG00000148019	0.76
46	PHF20L1	ENSG00000129292	0.76
47	PCNXL4	ENSG00000126773	0.76
48	AKAP9	ENSG00000127914	0.75
49	MYO10	ENSG00000145555	0.74
50	MMS22L	ENSG00000146263	0.74
51	SLITRK5	ENSG00000165300	0.74
52	POU2F2	ENSG00000028277	0.73
53	RP1-78B3.1	ENSG00000279652	0.73
54	RPS6KA6	ENSG00000072133	0.73
55	BBX	ENSG00000114439	0.72
56	EMX2	ENSG00000170370	0.72
57	GPR155	ENSG00000163328	0.72
58	CELF5	ENSG00000161082	0.72
59	ABI2	ENSG00000138443	0.72
60	GK5	ENSG00000175066	0.72
61	NFIC	ENSG00000141905	0.71
62	TUBGCP4	ENSG00000137822	0.71
63	MAVS	ENSG00000088888	0.71
64	PPP2R5E	ENSG00000154001	0.71
65	GABPB2	ENSG00000143458	0.71
66	FAM155A	ENSG00000204442	0.71
67	RAB21	ENSG00000080371	0.71
68	ELAVL4	ENSG00000162374	0.71
69	CNTLN	ENSG00000044459	0.70
70	SYNE2	ENSG00000054654	0.70
71	SOX4	ENSG00000124766	0.70
72	CH507-338C24.1	ENSG00000277991	0.70
73	PEX26	ENSG00000215193	0.70
74	NFIB	ENSG00000147862	0.70
75	RPAP2	ENSG00000122484	0.70
76	CCDC30	ENSG00000186409	0.70
77	SSTR2	ENSG00000180616	0.69
78	IGF1	ENSG00000017427	0.69
79	NF1	ENSG00000196712	0.69
80	ICE2	ENSG00000128915	0.69
81	FAM126A	ENSG00000122591	0.68
82	BAZ2B	ENSG00000123636	0.68
83	ZC3H8	ENSG00000144161	0.68
84	RASAL2	ENSG00000075391	0.68
85	RP11-717F1.2	ENSG00000274333	0.68
86	CTA-243E7.4	ENSG00000279085	0.68
87	ZFHX3	ENSG00000140836	0.68
88	GTF2H5	ENSG00000272047	0.68
89	NEUROD1	ENSG00000162992	0.68
90	PRR26	ENSG00000180525	0.68
91	DBT	ENSG00000137992	0.68
92	NEUROD2	ENSG00000171532	0.68
93	ZCCHC11	ENSG00000134744	0.67
94	TMEM19	ENSG00000139291	0.67
95	CUX1	ENSG00000257923	0.67
96	ZBTB7A	ENSG00000178951	0.66
97	TMEM192	ENSG00000170088	0.66
98	ZKSCAN1	ENSG00000106261	0.66
99	PCM1	ENSG00000078674	0.66
100	LRRIQ3	ENSG00000162620	0.66

Table S2. The top 100 human genes whose poly(dT)-based RNA sequencing expression quantification is predicted to be most likely affected by internal priming. The score represents the proportion of exonic alignments predicted by the linear model to originate from internal priming. The complete list of all ranked human genes, including their exonic length and A-SNR content, is available online: https://svoboda.shinyapps.io/SNRtable/.

REFERENCES

1. Wu, G., Schmid, M., Rib, L., Polak, P., Meola, N., Sandelin, A., and Jensen, T. H. (2020) A Two-Layered Targeting Mechanism Underlies Nuclear RNA Sorting by the Human Exosome. *Cell Reports,* **30**(7), 2387–2401.e5.
2. Ma, F., Fuqua, B. K., Hasin, Y., Yukhtman, C., Vulpe, C. D., Lusis, A. J., and Pellegrini, M. (2019) A comparison between whole transcript and 3’ RNA sequencing methods using Kapa and Lexogen library preparation methods. *BMC Genomics,* **20**(1), 9.
3. Ding, J., Adiconis, X., Simmons, S. K., Kowalczyk, M. S., Hession, C. C., Marjanovic, N. D., Hughes, T. K., Wadsworth, M. H., Burks, T., Nguyen, L. T., *et al.* (2020) Systematic comparison of single-cell and single-nucleus RNA-sequencing methods. *Nature Biotechnology,* **38**(6), 737–746.
